# P-278. Emerging Threat: Rising Incidence and Antimicrobial Resistance of Serratia marcescens in a Guatemalan Intensive Care Unit

**DOI:** 10.1093/ofid/ofae631.481

**Published:** 2025-01-29

**Authors:** Andrea Barrios, Ana Elisa Rosales, Jarleny Orozco, Franco Pezzarossi, Laura Valenzuela, Luis Rodriguez, Diego Erdmenger

**Affiliations:** Hospital General San Juan de Dios, Guatemala, Suchitepequez, Guatemala; Hospital General San Juan de Dios, Guatemala, Suchitepequez, Guatemala; Hospital General San Juan de Dios, Guatemala, Suchitepequez, Guatemala; Hospital General San Juan de Dios, Guatemala, Suchitepequez, Guatemala; Hospital General San Juan de Dios, Guatemala, Suchitepequez, Guatemala; Hospital General San Juan de Dios, Guatemala, Suchitepequez, Guatemala; Hospital General San Juan de Dios, Guatemala, Suchitepequez, Guatemala

## Abstract

**Background:**

*Serratia marcescen*, a gram-negative bacillus, is an important pathogen associated with hospital acquired infections. It ranks as the 5^th^most common Gram-negative organism in Intensive Care Units infections globally. With a global case-mortality of 2.8%, Serratia marcescens poses a substantial threat to healthcare systems.

The use of broad-spectrum antibiotics has led to increased antimicrobial resistance among Serratia strains. Rates of ESBL-producing isolates of Serratia have been reported ranging from 6-24% of all strains, in low- and middle-income countries.

This study evaluates the incidence and resistance patterns of Serratia isolates in Guatemala City.

**Methods:**

An ecological study was conducted at an intensive care unit from a third-level reference hospital in Guatemala City from 2022 to 2023. All microbiologic isolates of Serratia Marcescens were included, and antimicrobial resistance was determined through the use VITEK® MS (Maldi Tof) and VITEK® 2 systems. Antimicrobial resistance patterns were identified according to the Clinical and Laboratory Standard Institute (CLSI) guidelines. GyrA/ParC phenotype was inferred through resistance to fluoroquinolones.

**Results:**

A total of 20 isolates of Serratia were identified in 2022 and 49 in 2023. The majority of strains were isolated from respiratory samples (81.15%). There was a significant increase in Serratia positivity rates (4.72 vs 9.06 per 1000 cultures) and incidence of Serratia isolates (24.75 vs. 94.59 per 1000 admissions) from 2022 to 2023.

An increase in antimicrobial resistance was observed between 2022 and 2023. In 2022, 70% of strains where susceptible, and 20% showed only one mechanism of resistance. Conversely, in 2023, only 6% of strains where susceptible and over 57% presented more than one mechanism of resistance. An increase in inferred GyrA/ParC (5% vs 83.6%) and confirmed ESBL (0% vs 36.7%) phenotypes were observed.

**Conclusion:**

An increased use of antimicrobials in the ICU may be associated with the sudden and alarming rise in resistance in Serratia isolates. The clinical impact of these ecological changes is yet to be determined.

The escalating incidence and resistance of S. marcescen in Guatemala City highlights the urgency for enhanced surveillance and antimicrobial stewardship programs.

**Disclosures:**

**All Authors**: No reported disclosures

